# p53 Ubiquitination Comediated by HUWE1 and TRAF6 Contributes to White Spot Syndrome Virus Infection in Crustacean

**DOI:** 10.1128/jvi.02029-21

**Published:** 2022-03-23

**Authors:** Yi Gong, Tongtong Kong, Jude Juventus Aweya, Hongyu Ma, Yueling Zhang, Shengkang Li

**Affiliations:** a Guangdong Provincial Key Laboratory of Marine Biology, Shantou University, Shantou, China; b Southern Marine Science and Engineering Guangdong Laboratory, Guangzhou, China; c Institute of Marine Sciences, Shantou University, Shantou, China; d STU-UMT Joint Shellfish Research Laboratory, Shantou University, Shantou, China; University of Illinois at Urbana Champaign

**Keywords:** *Scylla paramamosain*, WSSV, p53 ubiquitination, apoptosis, ROS

## Abstract

p53, the guardian of the genome, is a short-lived protein that is tightly controlled at low levels by constant ubiquitination and proteasomal degradation in higher organisms. p53 stabilization and activation are early crucial events to cope with external stimuli in cells. However, the role of p53 ubiquitination and its relevant molecular mechanisms have not been addressed in invertebrates. In this study, our findings revealed that both HUWE1 (HECT, UBA, and WWE domain-containing E3 ubiquitin-protein ligase 1) and TRAF6 (tumor necrosis factor receptor-associated factor 6) could serve as E3 ubiquitin ligases for p53 in mud crabs (Scylla paramamosain). Moreover, the expression of HUWE1 and TRAF6 was significantly downregulated during white spot syndrome virus (WSSV) infection, and therefore the ubiquitination of p53 was interrupted, leading to the activation of apoptosis and reactive oxygen species (ROS) signals through p53 accumulation, which eventually suppressed viral invasion in the mud crabs. To the best of our knowledge, this is the first study to reveal the p53 ubiquitination simultaneously induced by two E3 ligases in arthropods, which provides a novel molecular mechanism of invertebrates for resistance to viral infection.

**IMPORTANCE** p53, which is a well-known tumor suppressor that has been widely studied in higher animals, has been reported to be tightly controlled at low levels by ubiquitin-dependent proteasomal degradation. However, recent p53 ubiquitination-relevant research mainly involved an individual E3 ubiquitin ligase, but not whether there exist other mechanisms that need to be explored. The results of this study show that HUWE1 and TRAF6 could serve as p53 E3 ubiquitin ligases and synchronously mediate p53 ubiquitination in mud crabs (Scylla paramamosain), which confirmed the diversity of the p53 ubiquitination regulatory pathway. In addition, the effects of p53 ubiquitination are mainly focused on tumorigenesis, but a few are focused on the host immune defense in invertebrates. Our findings reveal that p53 ubiquitination could affect ROS and apoptosis signals to cope with WSSV infection in mud crabs, which is the first clarification of the immunologic functions and mechanisms of p53 ubiquitination in invertebrates.

## INTRODUCTION

The tumor suppressor protein p53 is a redox-active transcription factor, involving diverse cellular processes, including apoptosis and reactive oxygen species (ROS), to cope with various stimuli that lead to genomic instability (1, 2). ROS, the cell’s products or by-products, can function as signaling molecules essential in redox signaling in cells (3), but the excess of ROS would cause cell death (4). The first evidence of p53-mediated apoptosis was from a study published in 1991, which reported that p53 could dramatically decrease cell viability and induce multiple apoptotic hallmarks in myeloid leukemia cells (5). Some studies also suggested that p53 could regulate the expression, cellular localization, and activation of key effectors relevant to the apoptosis process (6). In this case, the level and activity of p53 are tightly controlled in cells. Ubiquitination is a highly selective protein degradation approach that mediates the elimination of abnormal proteins, controls the half-lives of specific proteins by posttranslational modification (7, 8), and plays a pivotal role in protein homeostasis modulation. Ubiquitin-activating enzymes (E1s), ubiquitin-conjugating enzymes (E2s), and ubiquitin ligases (E3s) work in concert to regulate the process. Among them, E3s are the key factors in ubiquitin-mediated events, specifically recognizing the substrates (9). So far, the function and mechanism of p53 ubiquitination have been extensively studied in mammals: for example, MDM2 (murine double minute 2)-mediated ubiquitination has been considered a classical tumorigenesis pathway (10). However, the role of p53 ubiquitination in invertebrates has not been explored.

HUWE1 (HECT, UBA, and WWE domain-containing E3 ubiquitin-protein ligase 1), also known as Mule, ARF-BP1, E3 histone, UREB1, HECTH9, and LASU1, is an HECT E3 ubiquitin ligase for p53 in mammals (11). HUWE1 is highly conserved in mammals and was originally identified as a major binding protein associated with the ARF (ADP-ribosylation factor) tumor suppressor (12). HUWE1 has been reported to be highly expressed in colorectal, lung, stomach, ovarian, and breast carcinoma (11) and able to inhibit tumor growth by mediating the degradation of the Myc/Miz complex in a mouse skin cancer model (13). Besides, HUWE1 could ubiquitinate histone H1.3 to promote the transformation of ovarian epithelial cells and the development of ovarian cancer (14). In recent years, several studies have revealed that HUWE1 is tightly associated with proliferation/differentiation, p53-dependent apoptosis, and DNA repair in mammals (15), but its roles in the regulation of p53 ubiquitination remain poorly understood.

TRAF6 (tumor necrosis factor receptor [TNFR]-associated factor 6) is a crucial signaling molecule that regulates a wide range of physiological processes (16). Except for mediating TNFR family signaling pathways, TRAF6 appears to promote kinase activation through nondegradation ubiquitination of itself and downstream signaling molecules (17). Moreover, TRAF6 can regulate apoptosis by acting as an E3 ubiquitin ligase for p53 and can restrict p53 mitochondrial translocation through promoting K63-linked ubiquitination of p53 at K24 in the cytosol, which can further limit the interaction between p53 and the MCL-1/BAK complex (18). It has also been reported that TRAF6 could suppress p53 translocation to mitochondria and further regulate cell proliferation in cholangiocarcinoma (19). However, in invertebrates, whether TRAF6 could mediate p53 ubiquitination has not been investigated.

Generally, innate immunity, including humoral and cellular immune responses, is the main approach for invertebrates to resist harmful microbes (20). It was found that p53 could regulate apoptotic activity and influence virus infection in Marsupenaeus japonicas (21). Besides, a study has also shown that p53 directly interacts with Dorsal and further regulates the NF-κB pathway to cope with virus infection in Litopenaeus vannamei (22). Ubiquitination of p53 is also essential for antiviral immunity in mammals. The previous study reported that Vpu (HIV-1 viral protein U) interacts with the SCF (Skp1-cullin-F-box) complex and further inhibits the ubiquitination and proteasomal degradation of p53 protein in a β-TrcP-dependent manner, resulting in the activation of p53/Bax proteins and p53-mediated cell death in human T-lymphoblast cells (23). To explore the mechanism and immunological function of p53 ubiquitination in invertebrates, E3 ubiquitin ligase-mediated p53 ubiquitination was characterized in mud crabs (Scylla paramamosain). White spot syndrome virus (WSSV), an enveloped double-stranded DNA viral pathogen of marine crustaceans that has been widely used in the mud crab infection model (24, 25), was used in this study. The results of this study revealed that the downregulation of E3 ubiquitin ligase (HUWE1 and TRAF6) in mud crabs during WSSV infection resulted in the accumulation of p53 protein, eventually activating apoptosis and ROS signals in resistance to virus infection.

## RESULTS

### p53 ubiquitination is suppressed during WSSV infection in mud crabs.

**FIG 1:**
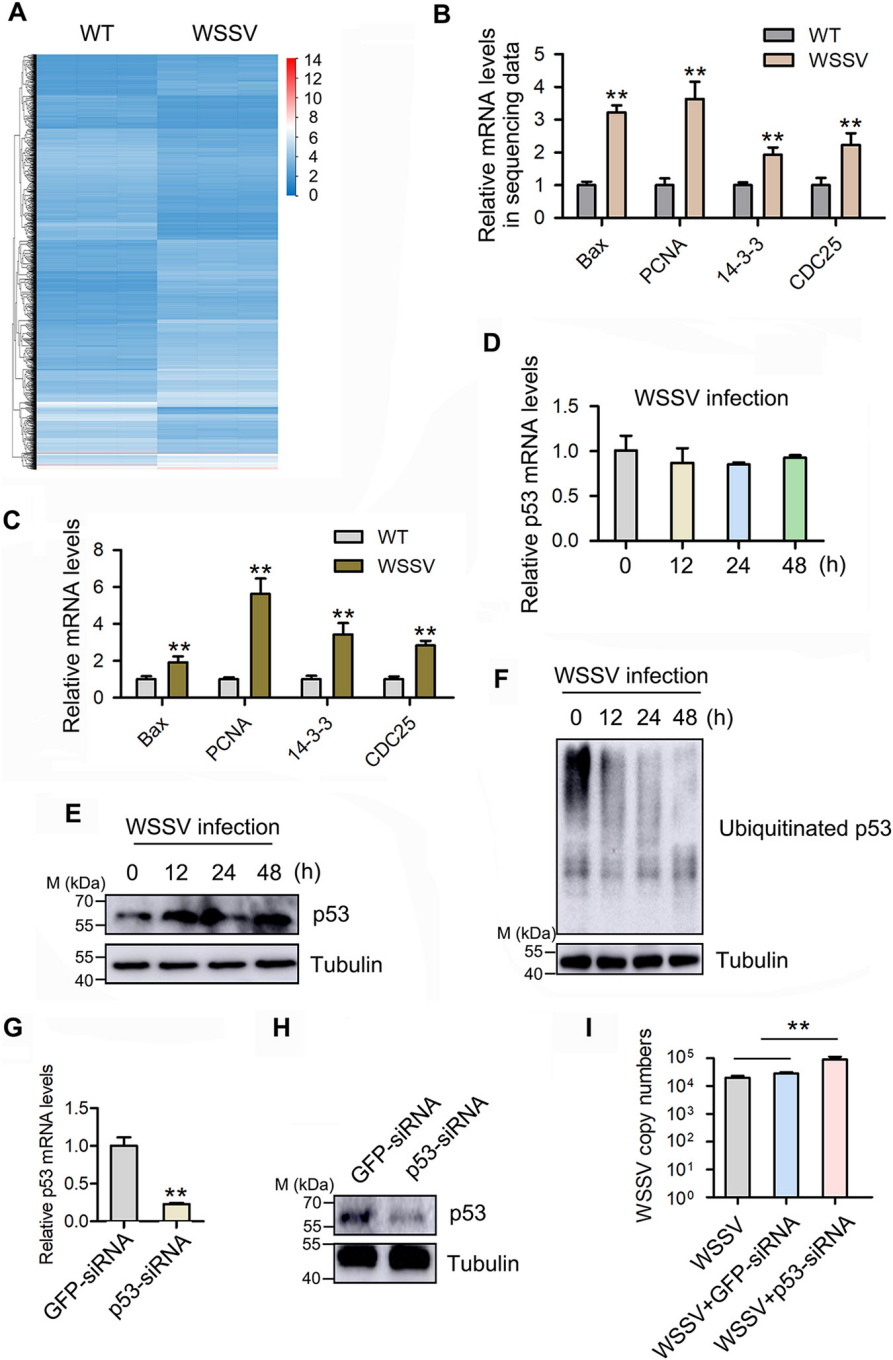
p53 ubiquitination is inhibited to cope with WSSV infection in mud crabs. (A). RNA-seq analysis of mud crabs challenged with WSSV infection. Mud crabs were injected with WSSV for 24 h, then the total RNA was isolated and subjected to RNA-seq by the Beijing Genomics Institute. WT, wild type. (B) mRNA expression of p53 downstream genes (coding for Bax, PCNA, 14-3-3, and CDC25) in sequencing data. (C) Validation of sequencing data shown in panel B via qPCR. (D and E) The expression of p53 in mud crabs during WSSV infection. Mud crabs were challenged with WSSV infection, and at 0, 12, 24, and 48 h postinfection, the mRNA (D) and protein (E) levels of p53 were detected through qPCR and Western blotting, respectively. (F) The influence of WSSV infection on p53 ubiquitination. Mud crabs were injected with WSSV, then the ubiquitination of p53 was detected at 0, 12, 24 and 48 h postinfection. (G and H) p53 interference efficiency detection. Mud crabs were treated with p53 siRNA and green fluorescent protein (GFP) siRNA for 48 h, respectively, then p53 mRNA levels (G) and protein levels (H) were measured by qPCR and Western blotting, respectively. (I) The effect of p53 silencing on WSSV infection. Mud crabs were treated with WSSV and p53 siRNA or GFP siRNA for 48 h, then WSSV copy numbers were measured. All data are represented as the mean ± standard deviation (SD) from three independent experiments (**, *P* < 0.01).

To explore the molecular mechanism of the innate immune response during virus infection in marine invertebrates, WSSV-challenged mud crabs were subjected to transcriptome sequencing (RNA-seq) (Fig. 1A), and the data were uploaded to the NCBI BioProject database. The RNA-seq data revealed that p53 downstream genes were remarkably upregulated during WSSV infection in the mud crabs (Fig. 1B), and the results were further confirmed by quantitative PCR (qPCR) (Fig. 1C), indicating that the p53 regulatory pathway was activated. To confirm this conjecture, p53 was detected during WSSV infection in mud crabs, and the results revealed that p53 remained unchanged at the mRNA level at 48 h postinfection (Fig. 1D), while at the protein level, p53 had accumulated since 12 h postinfection (Fig. 1E). We hypothesized that this result might be caused by the dysregulation of p53 ubiquitination. Therefore, we injected mud crabs with WSSV and detected p53 ubiquitination, and the results indicated that the ubiquitination of p53 was inhibited during virus infection (Fig. 1F). To further reveal the immunological significance of p53 accumulation in the mud crab, its expression was silenced (Fig. 1G and H), and the results demonstrated that the silencing of p53 significantly contributes to the WSSV infection (Fig. 1I), indicating that it could suppress viral infection. Taken together, the above findings suggested that p53 ubiquitination was inhibited during WSSV infection, resulting in the accumulation of p53 protein in the mud crab to cope with viral infection.

### p53 directly interacts with HUWE1 and TRAF6.

**FIG 2:**
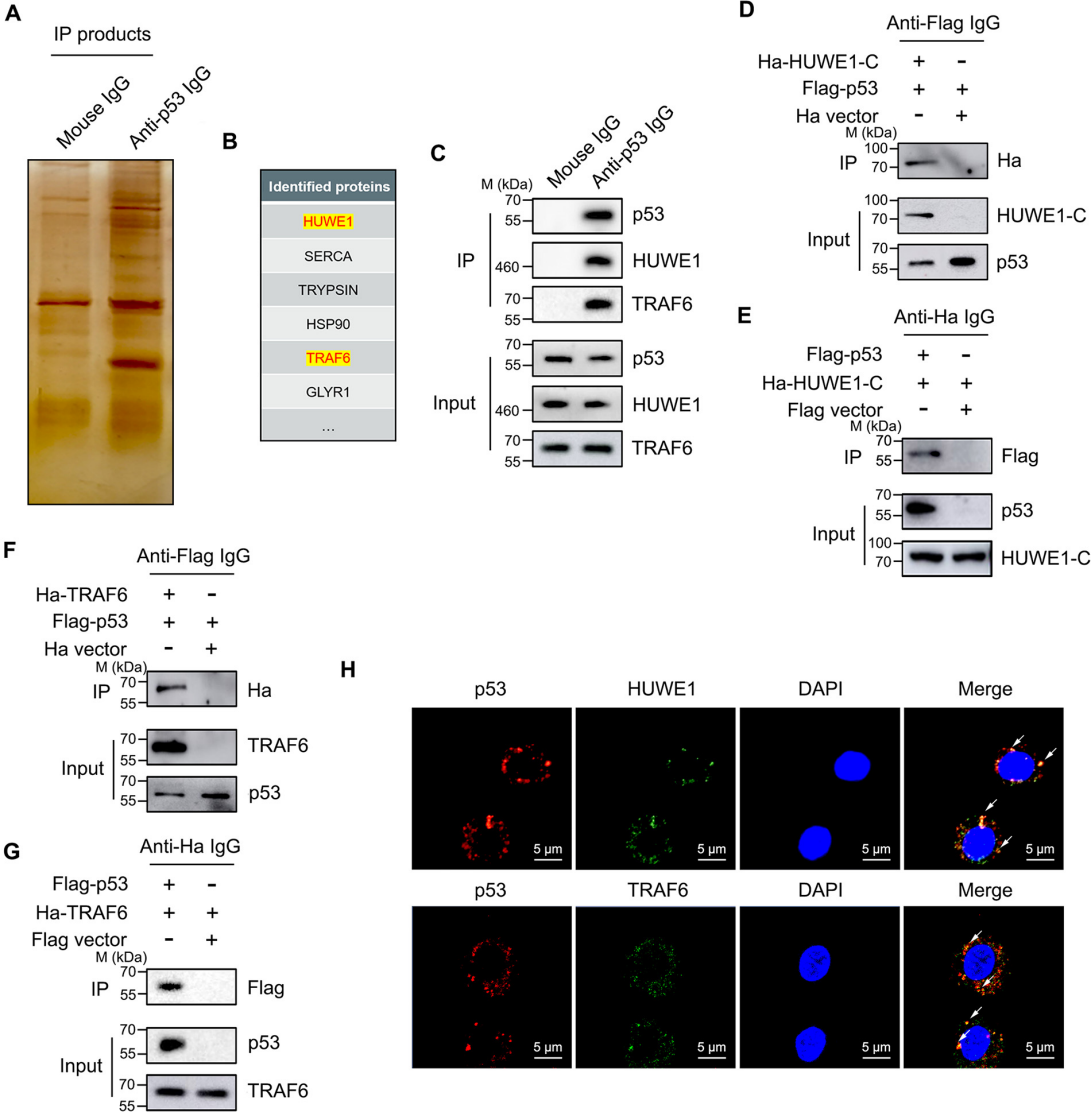
HUWE1 and TRAF6 are p53 binding proteins. (A and B) Identification of p53 binding proteins. The lysates of crab hemocytes were subjected to co-IP analysis using anti-p53 IgG, then the products were separated through SDS-PAGE (A) and identified by mass spectrometry (B). (C) The interactions between p53 and HUWE1 or TRAF6 in mud crabs. The lysates of crab hemocytes were subjected to co-IP assay with anti-p53 IgG, and then the IP products were subjected to Western blot analysis to detect HUWE1 and TRAF6. (D and E) The interactions between p53 and HUWE1 *in vitro*. Flag-tagged p53 and HA-tagged HUWE1-C plasmids were transfected into S2 cells for 48 h, then cell lysates were subjected to co-IP analysis using anti-Flag IgG (D) or anti-HA IgG (E), followed by Western blot analysis to detect HA or Flag tag. (F and G) The interactions between p53 and TRAF6 *in vitro*. Flag-tagged p53 and HA-tagged TRAF6 plasmids were transfected into S2 cells for 48 h, then cell lysates were subjected to co-IP analysis using anti-Flag IgG (F) or anti-HA IgG (G), followed by Western blot analysis to detect HA or Flag tag. (H) An immunofluorescence assay was performed to detect the localization of p53, HUWE1 and TRAF6 proteins in crab hemocytes, DAPI was used to stain the nucleus; the arrows indicate colocalization. Scale bar, 5 μm. Each experiment includes three independent biological repetitions.

In order to reveal the regulatory mechanism of p53 ubiquitination in the mud crab, pulldown analysis based on p53 was carried out, and the immunoprecipitation (IP) products were further subjected to SDS-PAGE and liquid chromatography-tandem mass spectrometry (LC-MS/MS) analysis (Fig. 2A). The identified proteins that only exist in the anti-p53 IgG group but not in the mouse IgG group were considered the potential interacting proteins of p53. The results showed that E3 ubiquitin ligase HUWE1 and TRAF6 might interact with p53 protein (Fig. 2B); more detailed information on p53 interacting proteins is shown in Table S1 in the supplemental material. Besides, the results of Western blot analysis also suggested that p53 could bind to HUWE1 and TRAF6 (Fig. 2C). To confirm these findings, Flag-tagged p53 and hemagglutinin (HA)-tagged HUWE1-C (HECT domain) plasmids were cotransfected into S2 cells; coimmunoprecipitation (co-IP) results showed that HA-HUWE1-C (HECT domain) and Flag-p53 were able to bring each other down (Fig. 2D and E), which demonstrated the interactions between HUWE1 and p53. Similarly, the same trends were observed by conducting co-IP analysis between p53 and TRAF6 (Fig. 2F and G). The above findings strongly indicated that p53 could bind to both HUWE1 and TRAF6 *in vitro*. To further confirm the direct interaction between p53 and HUWE1 or TRAF6 *in vivo*, the cellular distributions of p53, HUWE1, and TRAF6 proteins were observed under confocal microscopy, and the immunofluorescence images revealed that p53 protein was colocalized with HUWE1 and TRAF6 in the cytoplasm of mud crab hemocytes (Fig. 2H). Taken together, these data suggested that p53 could bind to E3 ubiquitin ligases HUWE1 and TRAF6, respectively, in the mud crab.

### HUWE1 and TRAF6 serve as E3 ubiquitin ligases for p53.

**FIG 3:**
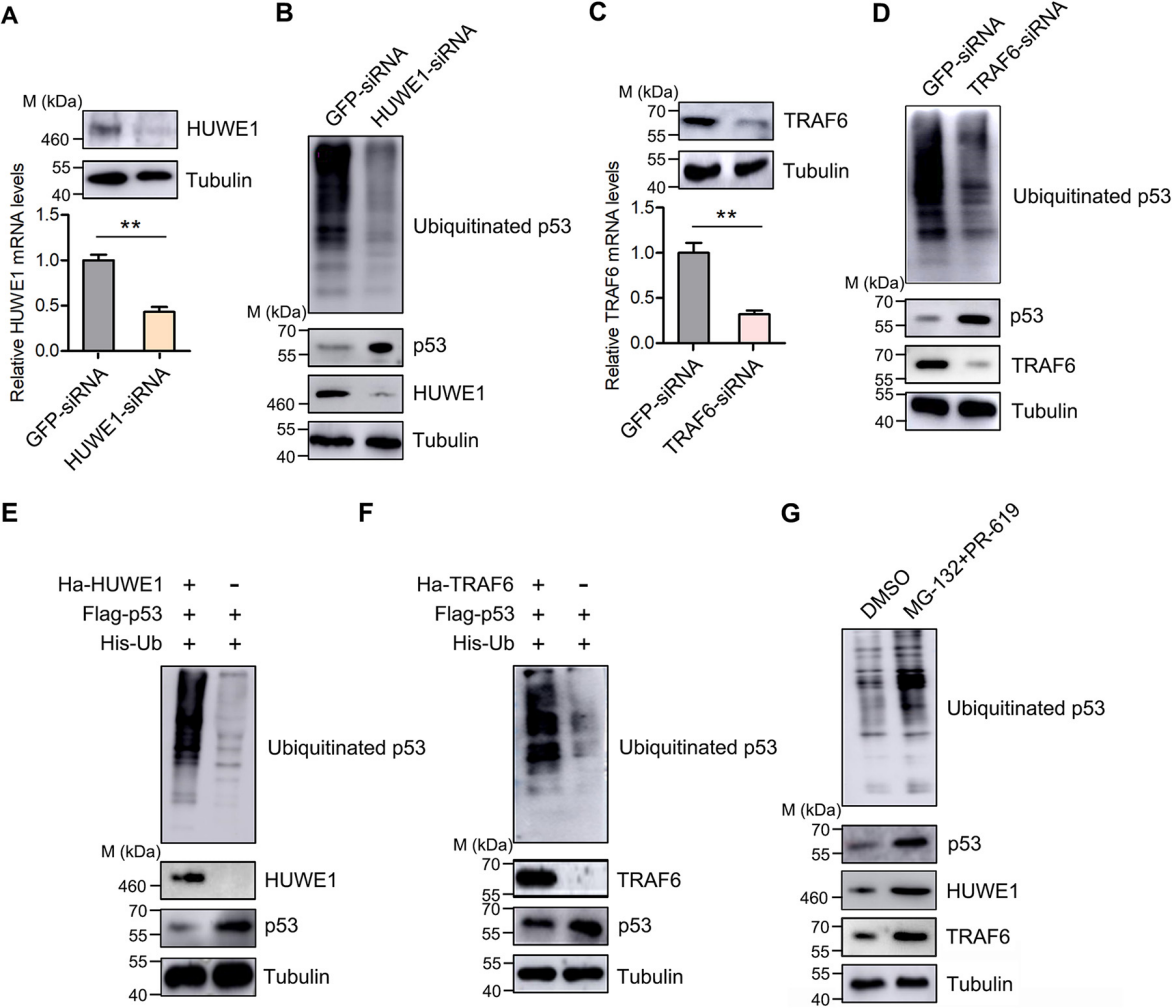
Both HUWE1 and TRAF6 are required for p53 ubiquitination. (A) HUWE1 interference efficiency detection. Mud crabs were treated with HUWE1 siRNA or GFP siRNA for 48 h, and then the mRNA and protein levels of HUWE1 were measured by qPCR and Western blotting, respectively. (B) The effects of HUWE1 silencing on the expression and ubiquitination levels of p53. (C) TRAF6 interference efficiency detection. Mud crabs were treated with TRAF6 siRNA or GFP siRNA for 48 h. Then, the mRNA and protein levels of TRAF6 were measured by qPCR and Western blotting, respectively. (D) The effects of TRAF6 silencing on the expression and ubiquitination levels of p53. (E) The effects of HUWE1 overexpression on p53 ubiquitination in mud crabs. p53, Ub, and/or HUWE1 was overexpressed in S2 cells with specific plasmids for 48 h, and then the expression and ubiquitination levels of p53 were evaluated. (F) The effects of TRAF6 overexpression on p53 ubiquitination in mud crabs. p53, Ub, and/or TRAF6 were overexpressed in S2 cells with specific plasmids for 48 h, and then the expression and ubiquitination levels of p53 were evaluated. (G) MG132 and PR-619 enhance the ubiquitination of p53 in mud crabs. Protease inhibitor MG132 and deubiquitination enzyme inhibitor PR-619 or DMSO was injected into mud crabs for 72 h, and then the expression and ubiquitination levels of p53 were evaluated. All of the numeral data represent the mean ± SD from triplicate assays (**, *P* < 0.01).

As with the previously reported E3 ubiquitin ligases (26, 27), whether HUWE1 and TRAF6 can mediate the ubiquitination of p53 in mud crabs remains unclear. To address this issue, HUWE1 and TRAF6 were silenced, and then the ubiquitination of p53 was evaluated. As shown in Fig. 3A and B, the depletion of HUWE1 would lead to the accumulation of p53 protein, and the ubiquitination of p53 was also reduced (Fig. 3B). In addition, p53 was accumulated when TRAF6 was suppressed (Fig. 3C and D); similarly, the ubiquitination of p53 was reduced (Fig. 3D). Moreover, the overexpression of HUWE1 and TRAF6 could promote the ubiquitination of p53 and lead to the reduction of p53 at the protein level in S2 (*Drosophila* Schneider 2) cells (Fig. 3E and F). To further explore whether p53 ubiquitination mediated by HUWE1 and TRAF6 was dependent on the proteasome pathway, we detected p53 ubiquitination in mud crabs treated with MG132 and PR-619 or dimethyl sulfoxide (DMSO). The results revealed that the presence of MG132 and PR-619 significantly increased the protein levels of both HUWE1 and TRAF6 (Fig. 3G). Moreover, we found that the increase of HUWE1 and TRAF6 led to the increase of p53 ubiquitination, while the amount of p53 protein did not decrease, but increased (Fig. 3G), indicating that p53 ubiquitination did depend on the proteasome pathway. Collectively, these data suggested that HUWE1 and TRAF6 could serve as E3 ubiquitin ligases and target p53 for ubiquitination and proteasomal degradation.

### p53 suppresses WSSV infection by activating apoptosis and ROS signals.

**FIG 4:**
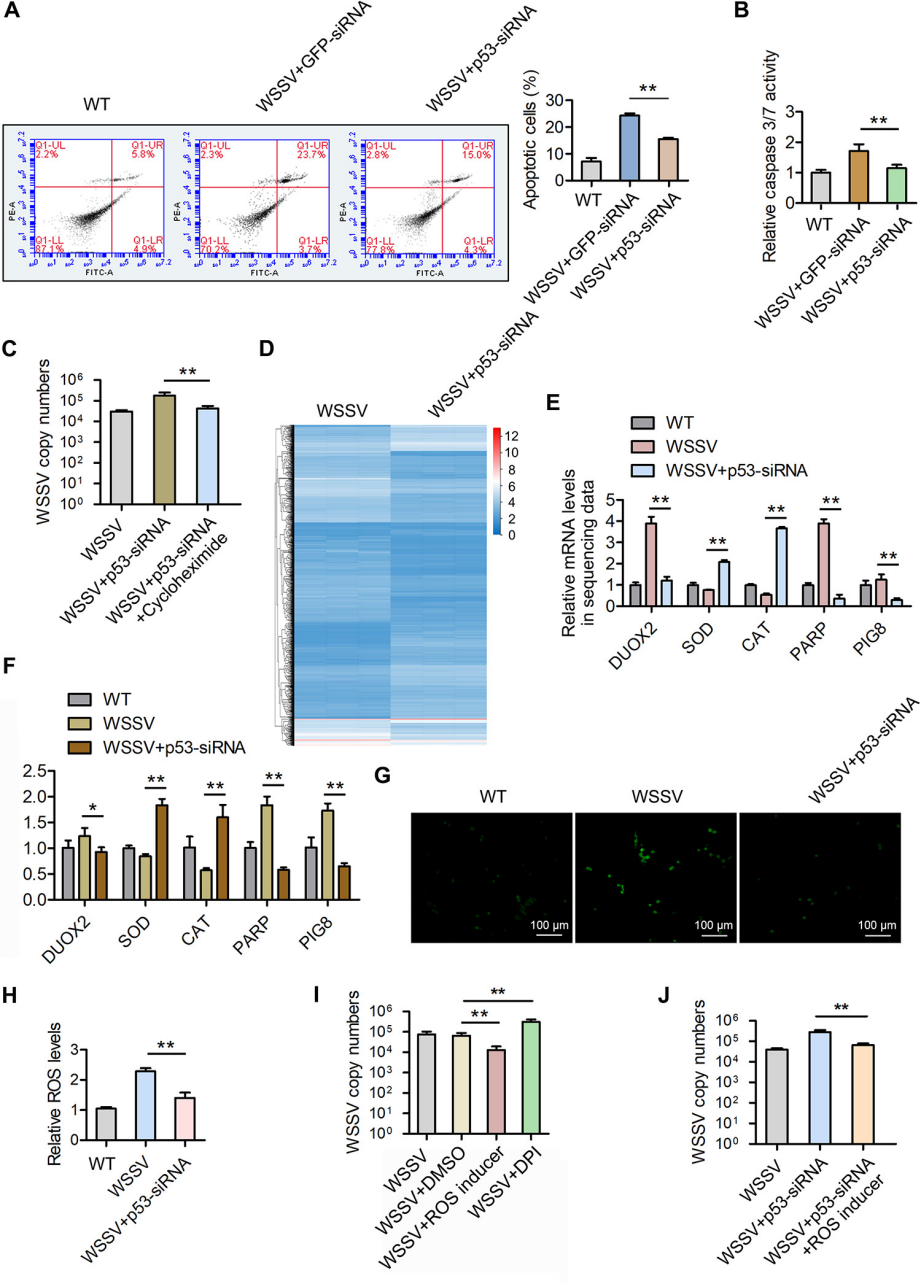
p53 activates apoptosis and ROS signals to suppress WSSV infection in mud crabs. (A and B) The effects of p53 silencing on apoptosis rate during WSSV infection in mud crabs. Mud crabs were coinjected with WSSV and p53 siRNA or GFP siRNA for 48 h, and then the hemocytes were collected and subjected to annexin V analysis (A) and caspase 3/7 activity detection (B). (C) The involvement of apoptosis during p53-mediated virus suppression. WSSV-challenged mud crabs were coinjected with p53 siRNA and/or the apoptosis inducer cycloheximide, followed by WSSV copy number detection. (D) RNA-seq analysis of mud crabs treated with p53 siRNA during WSSV infection. Mud crabs were injected with WSSV and p53 siRNA for 48 h, and then the total RNA was isolated and subjected to RNA-seq analysis. (E) The mRNA expression of ROS-associated genes (coding for DUOX2, SOD, CAT, PARP, and PIG8) in sequencing data. (F) Validation of sequencing data shown in panel E via qPCR. (G and H) The effects of p53 silencing on ROS production during WSSV infection in mud crabs. Mud crabs were coinjected with WSSV and p53 siRNA for 48 h, and then the hemocytes were collected and subjected to ROS measurement by fluorescence microscope (G) and microplate reader (H). (I) The influence of ROS levels on WSSV infection in mud crabs. WSSV-challenged mud crabs were treated with the ROS inhibitor DPI (Sigma-Aldrich, USA), ROS inducer (Bestbio, China), or DMSO for 48 h, followed by WSSV copy number detection. (J) The involvement of ROS during p53-mediated virus suppression in mud crabs. All data are the average from at least three independent experiments and are expressed as the mean ± SD (**, *P* < 0.01).

To reveal the molecular mechanism by which p53 regulates viral replication, we first detected apoptosis, the most well-known function of p53 (28). The results of annexin V and caspase 3/7 activity showed that silencing of p53 significantly reduced apoptosis activity in the mud crab (Fig. 4A and B). Besides, the p53 small interfering RNA (siRNA)-mediated virus replication promotion in mud crabs was restrained in the presence of the apoptosis inducer cycloheximide (Fig. 4C), suggesting that p53 could suppress WSSV infection by promoting apoptosis in the mud crab. As a multifunctional factor, whether p53 could regulate virus infection through other pathways needs to be addressed. Therefore, p53 was silenced followed by mRNA sequencing (Fig. 4D): the results showed that ROS-associated genes (coding for dual oxidase 2 [DUOX2], superoxide dismutase [SOD], catalase [CAT], poly ADP-ribose polymerase [PARP], and p53-inducible genes 8 [PIG8]) were dysregulated (Fig. 4E), which was also confirmed by qPCR (Fig. 4F). To assess whether p53 could affect ROS production, p53 was silenced, and then the level of ROS was evaluated, and the results showed that silencing of p53 would reduce ROS production in mud crabs (Fig. 4G and H). Moreover, the ROS inhibitor diphenyleneiodonium chloride (DPI) was found to promote WSSV replication, while ROS inducer could suppress WSSV replication (Fig. 4I), indicating that ROS possess negative effects on viral infection in mud crabs. In addition, we found that ROS inducer could suppress p53 silencing-caused virus replication promotion (Fig. 4J), suggesting that p53 could suppress WSSV infection by increasing ROS production. Taken together, the above data showed that p53 could activate apoptosis and ROS signals to cope with WSSV infection in mud crabs.

### HUWE1 and TRAF6 suppress apoptosis and ROS production by regulating p53.

**FIG 5:**
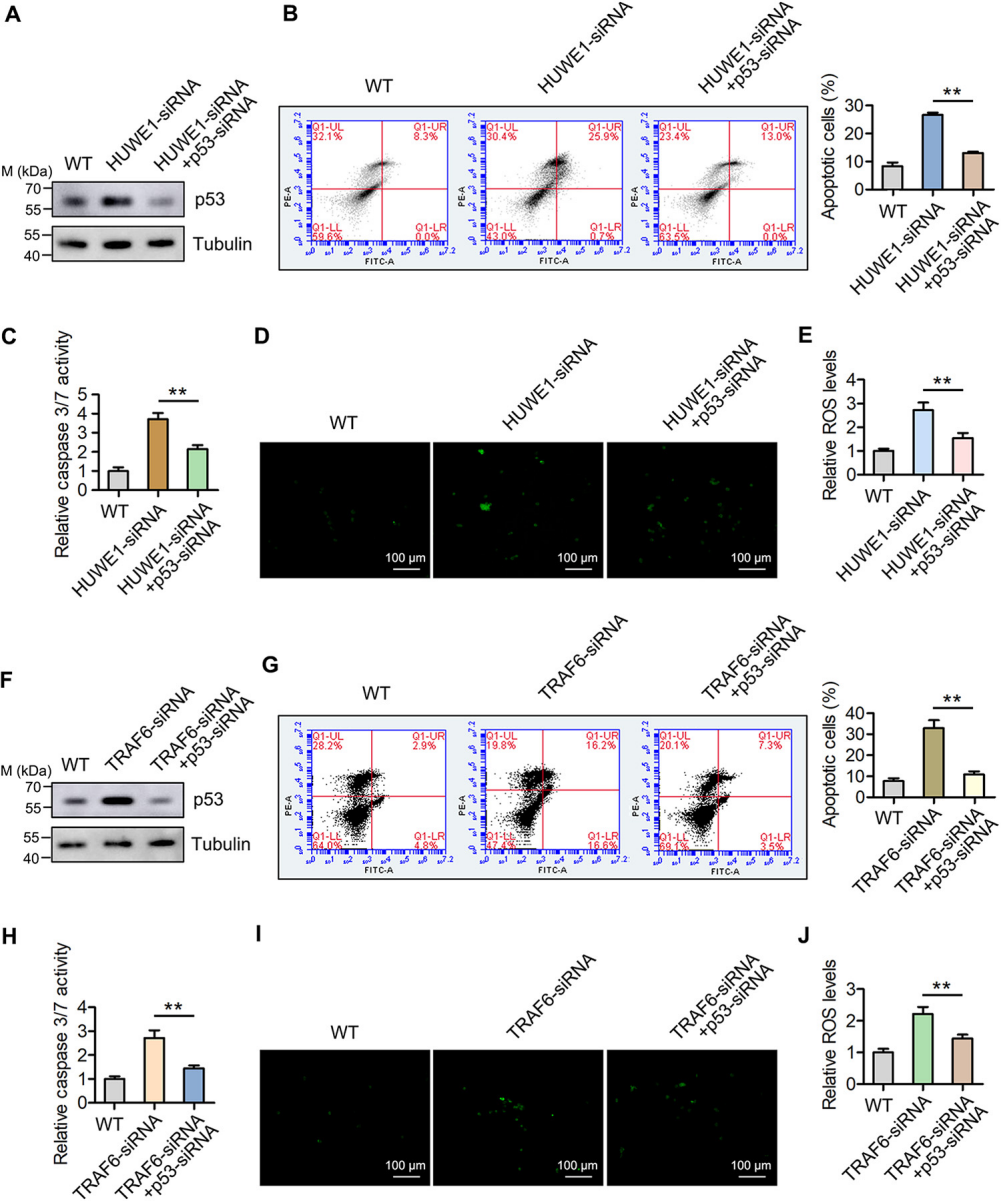
HUWE1 and TRAF6 serve as negative regulators for apoptosis and ROS signals by suppressing p53. (A) Western blot analysis of p53 protein in mud crabs after being cotreated with HUWE1 siRNA and p53 siRNA; tubulin was used as an internal reference. (B and C) HUWE1 suppresses apoptosis signals by regulating p53. Mud crabs were cotreated with HUWE1 siRNA and p53 siRNA for 48 h, followed by apoptosis evaluation via annexin V analysis (B) and caspase 3/7 activity detection (C). (D and E) HUWE1 suppresses ROS production by regulating p53. Mud crabs were cotreated with HUWE1 siRNA and p53 siRNA for 48 h, followed by ROS measurement using a fluorescence microscope (D) and microplate reader (E). (F) Western blot analysis of p53 protein in mud crabs after being cotreated with TRAF6 siRNA and p53 siRNA; tubulin was used as an internal reference. (G and H) TRAF6 suppresses apoptosis signals by regulating p53. Mud crabs were cotreated with TRAF6 siRNA and p53 siRNA for 48 h, followed by apoptosis evaluation via annexin V analysis (G) and caspase 3/7 activity detection (H). (I and J) TRAF6 suppresses ROS production by regulating p53. Mud crabs were cotreated with TRAF6 siRNA and p53 siRNA for 48 h, followed by ROS measurement using a fluorescence microscope (I) and microplate reader (J). Data represent the mean ± SD from triplicate assays (**, *P* < 0.01).

HUWE1 and TRAF6 are the E3 ubiquitin ligases for p53; therefore, whether they could regulate apoptosis and ROS production needs to be further explored. To address this issue, mud crabs were cotreated with HUWE1 siRNA and p53 siRNA (Fig. 5A), followed by the detection of apoptosis and ROS production. The data showed that silencing of HUWE1 resulted in the upregulated apoptosis levels by annexin V and caspase 3/7 activity detections (Fig. 5B and C), and this process was repressed by p53 interference (Fig. 5B and C). Furthermore, through ROS detection with a fluorescence microscope and microplate reader, we found that HUWE1 could remarkably suppress ROS production by regulating p53 (Fig. 5D and E). Similarly, by treating mud crabs with TRAF6 siRNA and p53 siRNA (Fig. 5F), we found that TRAF6 could significantly suppress apoptosis and ROS production by regulating p53 (Fig. 5G to J). Taken together, the above findings indicated that both HUWE1 and TRAF6 could suppress apoptosis and ROS production by regulating p53 in mud crabs.

### HUWE1 and TRAF6 contribute to WSSV infection in mud crabs.

**FIG 6:**
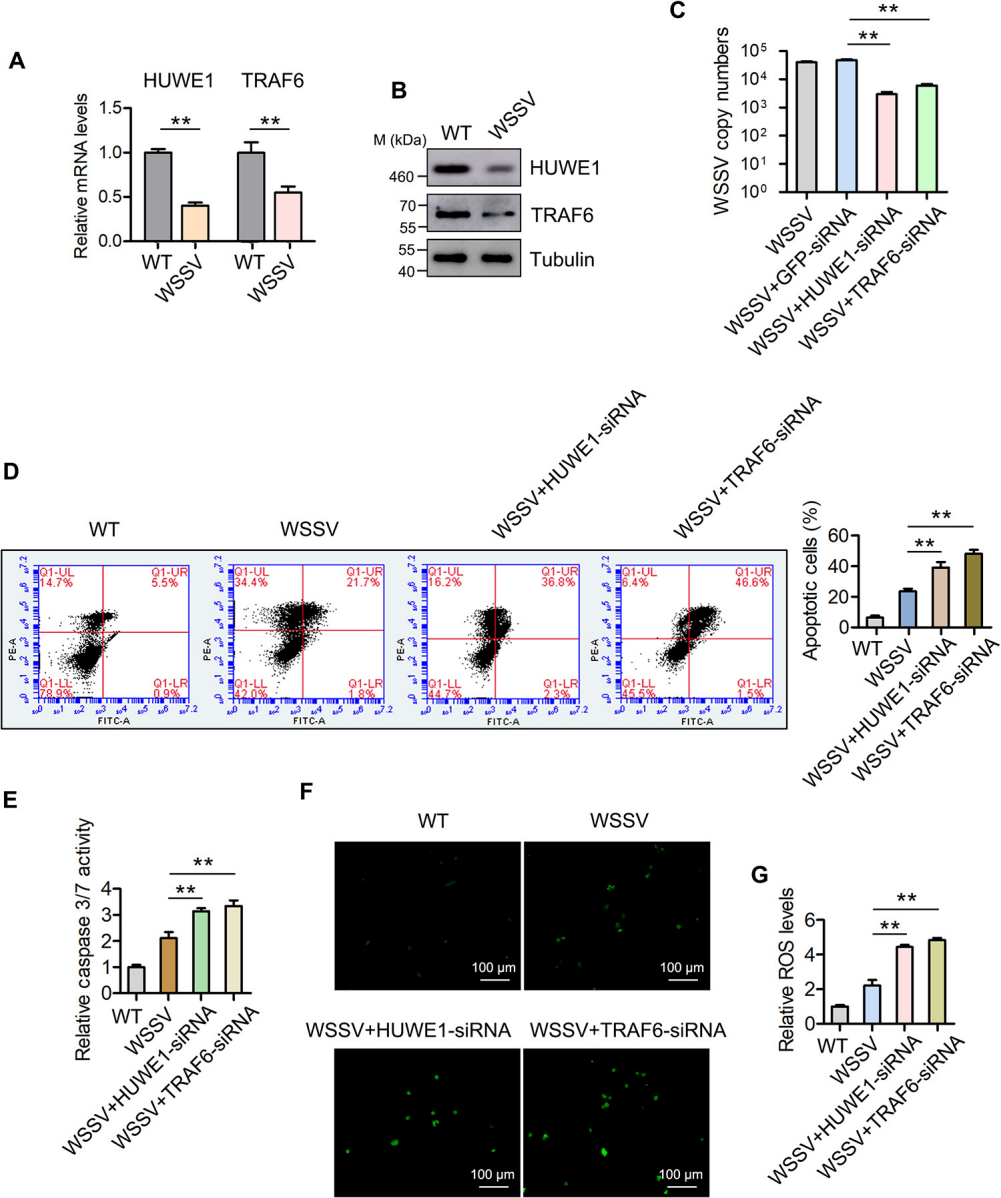
HUWE1 and TRAF6 serve as negative regulators for apoptosis and ROS signals by suppressing p53. (A) The influence of WSSV infection on HUWE1 and TRAF6 mRNA expression levels. Mud crabs were challenged with WSSV for 24 h, and then the mRNA levels of HUWE1 and TRAF6 were detected by qPCR. (B) The influence of WSSV infection on HUWE1 and TRAF6 protein levels in mud crabs. (C) The effects of HUWE1 and TRAF6 silencing on WSSV replication. Mud crabs challenged with WSSV infection were coinjected with HUWE1 siRNA or TRAF6 siRNA for 48 h, and then WSSV copy numbers were measured. (D and E) HUWE1 and TARF6 promote WSSV infection by suppressing apoptosis signals in mud crabs. Mud crabs challenged with WSSV infection were coinjected with HUWE1 siRNA or TRAF6 siRNA for 48 h, followed by apoptosis evaluation via annexin V analysis (D) and caspase 3/7 activity detection (E). (F and G) HUWE1 and TARF6 promote WSSV infection by inhibiting ROS production in mud crabs. Mud crabs challenged with WSSV were coinjected with HUWE1 siRNA or TRAF6 siRNA for 48 h, followed by ROS measurement using a fluorescence microscope (F) and microplate reader (G). Asterisks indicate significant differences (**, *P* < 0.01).

To reveal the immunological significance of the HUWE1 and TRAF6 genes in regulating apoptosis and ROS signals, the relative expression of each of these genes was detected in mud crabs during WSSV infection. The results showed that at both mRNA and protein levels, HUWE1 and TRAF6 were decreased during the infection (Fig. 6A and B). Besides, silencing of HUWE1 or TRAF6 significantly suppressed the WSSV replication (Fig. 6C), indicating that these proteins could promote viral infection in the mud crab. In addition, we found that apoptosis and ROS levels were upregulated when HUWE1 and TRAF6 were silenced during WSSV infection (Fig. 6D to G), suggesting the regulation of HUWE1 and TARF6 in apoptosis and ROS production during the infection. Taken together, the results demonstrated that HUWE1 and TRAF6 were downregulated during WSSV infection, leading to the increase in apoptosis and ROS levels and resisting the virus infection in the mud crab.

**FIG 7:**
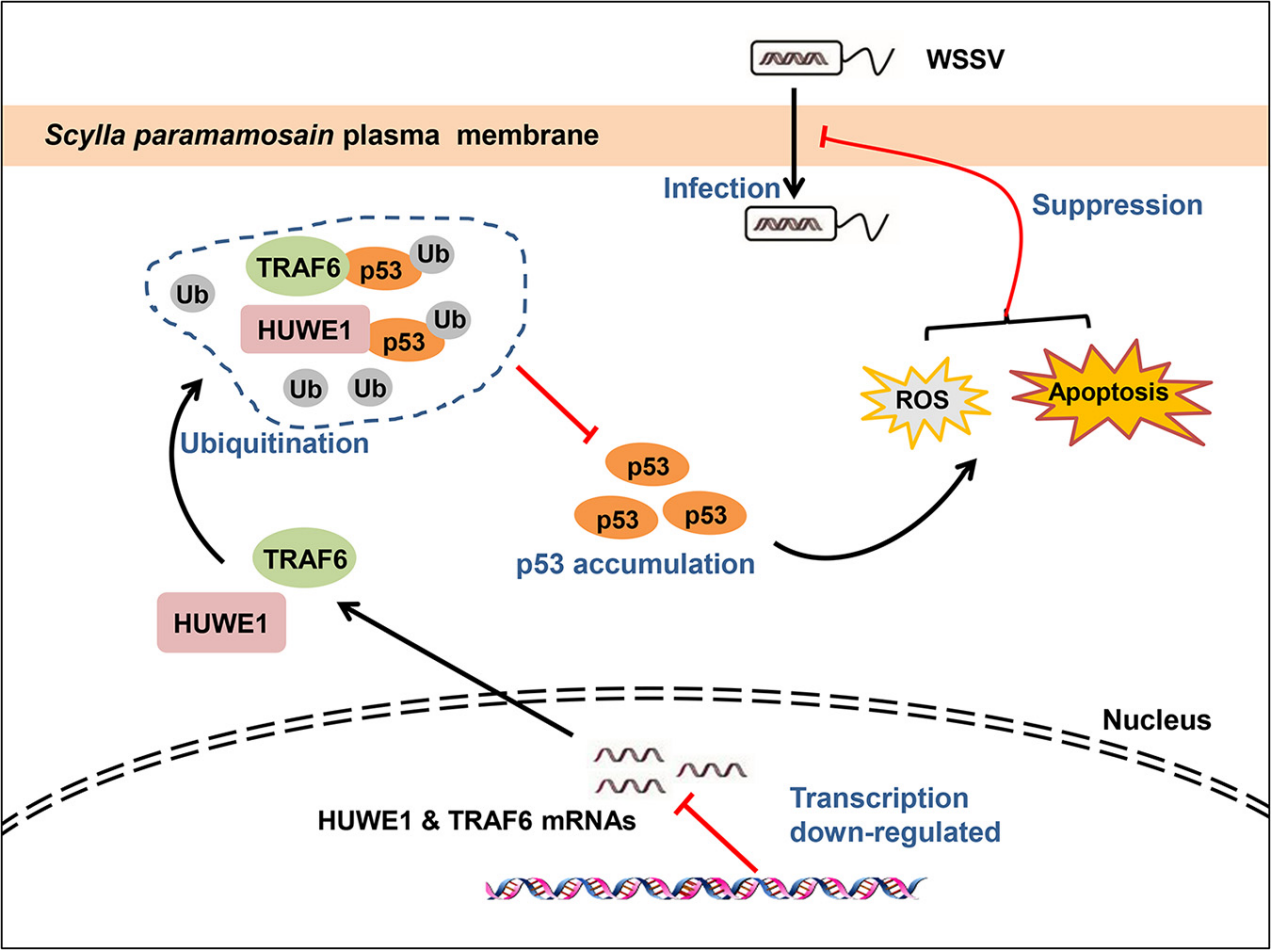
The proposed schematic diagram for p53 ubiquitination-mediated apoptosis, ROS, and regulation of virus invasion in mud crabs. During WSSV infection, the expression of HUWE1 and TRAF6 was suppressed: thus, the ubiquitination of p53 was interrupted, which further led to the accumulation of p53 protein, which eventually activated apoptosis and ROS signals to cope with virus invasion in the mud crabs.

To sum up, our findings showed that both HUWE1 and TRAF6 could serve as an E3 ubiquitin ligase for p53 and are downregulated in the mud crab during WSSV infection. Therefore, the ubiquitination of p53 is interrupted and further leads to the accumulation of p53, resulting in the activation of apoptosis and ROS signals to cope with virus invasion in the mud crab (Fig. 7).

## DISCUSSION

Ubiquitination is a kind of posttranslational modification by direct conjugation of ubiquitin molecules to the specific protein substrate via isopeptide bonds; the transfer of ubiquitin requires an enzymatic cascade (29). Among the enzymes, E3 ubiquitin ligases are responsible for protein substrate specificity (30) and have been proven to be the most abundant and diverse ubiquitin-associated enzymes, which are encoded by several hundred genes in mammalian genomes (31). p53 has been widely known as an important tumor suppressor through triggering cell apoptosis under environmental or oncogenic stress; loss or gain of p53 function would cause aberrant cell growth (32). Therefore, the protein level of p53 is tightly controlled in cells. MDM2 was the first identified p53 E3 ubiquitin ligase (33), followed by PIRH2 (ring finger and CHY zinc finger domain containing 1) (34), HUWE1, WWP1 (WW domain-containing E3 ubiquitin-protein ligase 1) (35), and COP1 (constitutively photomorphogenic 1) (36), which possess fundamental roles in the ubiquitin-dependent p53 protein degradation pathway. However, recent research indicated that p53 ubiquitination only involves an individual E3 ubiquitin ligase (37); however, whether there exist other mechanisms needs to be explored. In this study, we found that both HUWE1 and TRAF6 could serve as p53 E3 ubiquitin ligases and synchronously mediate p53 ubiquitination in mud crabs. To the best of our knowledge, this is the first report to confirm that p53 can be simultaneously regulated by two E3 ubiquitin ligases. This indicates the diversity of the p53 ubiquitination regulatory approach and provides novel insights into the innate immunity of invertebrates.

The functions and regulatory mechanisms of p53 (including the E3 ligase-mediated p53 ubiquitination) have been extensively investigated in the past few decades (38). The research has shown that p53 ubiquitination does not merely lead to protein degradation. For example, the MDM2-mediated p53 ubiquitin modification possesses multiple functions, including p53 degradation and mitochondrial translocation (39), while PIRH2 is relevant to the p53 degradation and tetramerization (40). The other two proteins (ICP0 and Ubc13) are not involved in p53 degradation and are related to the nucleus translocation and (41) tetramerization (42) of p53, respectively. At present, most of the studies relevant to p53 ubiquitination in higher animals have mainly focused on tumorigenesis, but a few have studied its effect on the host’s immune defense. It was reported that MeCP2 (methyl-CpG binding protein 2) could promote ubiquitination-dependent p53 degradation and further inhibit RPL5/RPL11 transcription, which eventually facilitates the carcinogenesis of breast cancer (43). Besides, RBCK1 (RANBP2-type and C3HC4-type zinc finger-containing 1) has been shown to directly interact with p53 protein and facilitate its polyubiquitination and degradation, restoring the function of p53 by RBCK1, which is considered a promising strategy for renal cell carcinoma therapy (44). In addition, the open reading frame 3 (ORF3) protein of porcine circovirus type 2 (PCV2) could bind to PIRH2 and suppress PIRH2-mediated p53 ubiquitination, resulting in the accumulation of p53 during viral infection, and the ORF3-induced apoptosis plays a major role in PCV2 pathogenesis (45). So far, p53 has been poorly studied in invertebrates. It was found that p53 could regulate Mn superoxide dismutase (MnSOD) and glutathione peroxidase (GPx) to cope with acute environmental stresses in shrimp (46). Few studies have shown that WSSV infection could activate the p53-dependent apoptotic pathway in shrimp (21, 22); however, p53 ubiquitination and its relevant mechanisms involved in the immune response against viral infection are still unknown. In our study, the mud crab was widely used as an invertebrate model for the WSSV infection. The results showed that the expression of HUWE1 and TRAF6 was decreased during WSSV infection, leading to the interruption of p53 ubiquitination, and therefore, p53 accumulated to cope with viral invasion in the mud crab. Our work is the first to reveal the functions and mechanisms of p53 ubiquitination in invertebrates, suggesting the conservation of the p53 ubiquitination pathway in animals and its essential roles in the antiviral immune regulation of marine invertebrates.

HUWE1 and TRAF6 are E3 ligases for p53 that have been confirmed to be closely associated with tumorigenesis (18, 26). HUWE1 could serve as a key factor associated with ARF in H1299 cells, which is crucial for both the p53-independent and p53-dependent tumor suppressor functions mediated by ARF through directly binding and ubiquitinating p53 (47). Besides, TRAF6 was found to suppress mitochondrial translocation of p53 and restrict spontaneous apoptosis by mediating K63-linked ubiquitination of p53 at K24 in U2OS cells (18). In recent years, the immunological functions of HUWE1 and TRAF6 have also been studied. For example, the classical swine fever virus (CSFV) nonstructural protein 3 (NS3) is a multifunctional protein required for viral replication. TRAF6 was found to suppress CSFV replication through interacting with NS3 (48). TRAF6 could protect FHM (Fathead minnow) cells from cell death induced by Singapore grouper iridovirus (SGIV) through repressing virus-induced production of type I interferons (IFNs) (49). Studies have also shown that HUWE1 could act as a DNA repair protein and help to maintain chromosomal nondisjunction in Caenorhabditis elegans under ionizing radiation (50). In addition, it was found that HUWE1 interacts with HIV-1 Gag-Pol precursor protein via the IN domain and has a negative impact on the next round of viral infection by regulating early postentry events (51). At present, whether the immunologic roles of HUWE1 and TRAF6 are related to the characteristics of E3 ligase has not been addressed. In our study, the results demonstrated that both HUWE1 and TRAF6 could regulate WSSV replication by promoting p53 ubiquitination and further affect ROS and apoptosis signals during WSSV infection in the mud crab. The above findings enrich our understanding of the immunological role of HUWE1 and TRAF6 and could be the basis of treatment strategies for viral diseases in the future.

## MATERIALS AND METHODS

### Mud crab culture and WSSV challenge.

Healthy mud crabs (approximately 50 g each) were purchased from Niutianyang (Shantou, Guangdong, China). Crabs were acclimated in water with 10‰ salinity at 25°C for a week before further processing. Then, 200 μL of WSSV suspension (1 × 10^6^ copies/mL) was injected into each crab through the base of the fourth leg. In the blank group, each crab was injected with 200 μL of phosphate-buffered saline (PBS). At different times postinjection, hemocytes and muscles of three randomly chosen crabs per group were collected as previously described (52) and stored at −80°C for later use.

### Quantification of mRNA with real-time PCR.

Total RNA was extracted from mud crab hemocytes by using TRIzol (Cwbio, Beijing, China) according to the manufacturer’s protocol, followed by cDNA synthesis with the QuantScript RT kit (Tiangen, China), and the cDNA was used as a template for qPCR using the SYBR green system (Tiangen, China). Gene-specific primers are listed as follows: HUWE1-F (5′-GGCTTATCTACCTGATGGAACACA-3′), HUWE1-R (5′-AGGAATATTGCGTCCGTTGG-3′), TRAF6-F (5′-CCAATTGACAA CACCCCTCTG-3′), TRAF6-R (5′-GGCGGAACACTCATTCGGAC-3′), p53-F (5′-CAGGAGGTGCTAATAAGGGTAACG-3′), and p53-R (5′-TTCACAACGATGGGAGGG GT-3′). The primers β-actin-F (5′-GCGGCAGTGGTCATCTCCT-3′) and β-actin-R (5′-GCCCTTCCTCACGCTATCCT-3′) were used to quantify the internal control (β-actin). Relative fold change was analyzed by the threshold cycle (2^−ΔΔ^*^CT^*) algorithm (53).

### Analysis of WSSV copies with quantitative real-time PCR.

The total DNA of WSSV-infected mud crabs was extracted with an SQ tissue DNA kit (Omega Bio-Tek, USA) according to the manufacturer’s instructions. Then, the WSSV copy numbers in the mud crabs were analyzed by qPCR with WSSV-specific TaqMan probe (5′-FAM [i.e., 6-carboxyfluorescein]-TGCTGCCGTCTCCAA-TAMRA [i.e., 6-carboxytetramethylrhodamine]-3′) and primers (F [forward], 5′-TTGGTTTCATGCCCGAGATT-3′; R [reverse], 5′-CCTTGGTCAGCCCCTTGA-3′). The PCR procedure was 95°C for 1 min, followed by 40 cycles of 95°C for 30 s, 52°C for 30 s, and 72°C for 30 s. The internal standard of qPCR was a DNA fragment of 1,400 bp from the WSSV genome, as previously described (54).

### RNA interference assay.

Double-stranded RNA duplexes composed of 21-nucleotide sense and antisense oligonucleotides were synthesized by an *in vitro* transcription T7 kit (TaKaRa, Japan). The RNA oligonucleotides used for targeting p53, HUWE1, and TRAF6 in this study are listed as follows: si-p53-1 (5′-GATCACTAATACGACTCACTATAGGGCCTAACAG CCATGTGCCTTTT-3′), si-p53-2 (5′-AAAAGGCACATGGCTGTTAGGCCCTATAGTGGATCGTATTAGTGATC-3′), si-p53-3 (5′-AACCTAACAGCCATGTGCCTTTTCCCTATAGTGAGTCGTATTAGTGATC-3′), si-p53-4 (5′-GATCACTAATACGACTCACTATAGGGTTCATAATCATCATCGTCCTT-3′), si-HUWE1-1 (5′-GATCACTAATACGACTCACTATAGGGGCACATCTCCATCATGAAATT-3′), si-HUWE1-2 (5′-AATTTCATGATGGAGATGTGCCCCTATAGTGAGTCGTATTAGTGATC-3′), si-HUWE1-3 (5′-AAGCACATCTCCATCATGAAACCCTATAGTGAGTCGTATTAGTGATC-3′), si-HUWE1-4 (5′-GATCACTAATACGACTCACTATAGGGTTTCATGATGGAGATGTGCTT-3′), si-TRAF6-1 (5′-GATCACTAATACGACTCACTATAGGGGCTTCTCCCAGCTTGCAATTT-3′), si-TRAF6-2 (5′-AAATTGCAAGCTGGGAGAAGCCCCTATAGTGAGTCGTATTAGTGATC-3′), si-TRAF6-3 (5′-AAGCTTCTCCCAGCTTGCAATCCCTATAGTGAGTCGTATTAGTGATC-3′), and si-TRAF6-4 (5′-GATCACTAATACGACTCACTATAGGGATTGCAAGCTGGGAGAAGCTT-3′). Then, 50 μg of the synthesized siRNA was injected into each mud crab, and 48 h postinfection, three mud crabs were randomly selected for each treatment and stored at −80°C for later use.

### RNA extraction, library construction, and RNA-seq.

Based on our previous studies (55, 56), the period 24 h postinfection was chosen for sampling in this study. Total RNA of hemolymph collected from mud crabs was isolated using TRIzol reagent (Ambion, USA) according to the manufacturer’s instructions. The integrity, concentration, and quality of the isolated RNAs were evaluated by NanoDrop 1000 spectrophotometer (Thermo Scientific, USA) and Agilent 2100 Bioanalyzer (Santa Clara, USA). Then, library construction and subsequent RNA-seq were conducted by Beijing Genomics Institute (BGI, Shenzhen, China) through the BGISEQ-500 sequencer. The data were uploaded to the NCBI BioProject database.

### Co-IP assay.

*Drosophila* Schneider 2 (S2) cells (Invitrogen), a cell line that is widely used in marine invertebrate-related research (57, 58), were cultured at 27°C in Express Five serum-free medium (SFM) (Invitrogen). Then, the constructed pIZ/V5 plasmid combinations bearing a Flag or HA tag were cotransfected into S2 cells using the Cellfectin II reagent (Invitrogen, USA). At 48 h after transfection, the cells were harvested and subjected to the co-IP analysis using the Pierce coimmunoprecipitation kit (Thermo Scientific, USA) following the manufacturer’s instructions. In brief, cells were lysed on ice with lysis buffer and then incubated with the resins at 4°C overnight. (The resins were coupled with the indicated antibodies in advance.) After that, the resins were washed three times with lysis buffer, followed by collection using elution buffer, and subjected to Western blotting. The primers used for plasmid construction are listed below: Ha-HUWE1 (F, 5′-TAGTCCAGTGTGGTGGAATTCATGTACCCATACGACGTCCCAGACTACGCTGCAGCAAGCAGCCAAGACC-3′; R, 5′-GAAGGGCCCTCTAGACTCGAGTTAGGCAAAGCCAAAGCCTTC-3′), Ha-TRAF6 (F, 5′-CTGATATCATGGCTTGCCACAATTCCCTC-3′; R, 5′-ATAAGAATGCGGCCGCTCAAGCGTAGTCTGGGACGTCGTATGGGTAAACCTTCTGAGATTTCTGCTGGTG-3′), and Flag-p53 (F, 5′-TAGTCCAGTGTGGTGGAATTCATGGATTACAAGGATGACGACGATAAGATGCGTCCAGCAACAAAGAGGT-3′; R, 5′-GAAGGGCCCTCTAGACTCGAGTTAAAGCTCATCTTCAGAAAACAG-3′).

### Western blotting.

The samples were mixed with 5× SDS loading buffer and separated by 12% SDS–polyacrylamide gel, and then transferred onto a nitrocellulose membrane (Millipore, USA). The membrane was blocked with QuickBlock Western (Beyotime, China) and further incubated with appropriate primary antibodies at 4°C overnight. After being washed three times with TBST (Tris-buffered saline plus Tween 20), the membrane was incubated with a secondary antibody for subsequent detection by ECL enhanced chemiluminescence substrate (Thermo Scientific, USA). Flag, His, HA, and histone H3 antibodies, Cy3-labeled goat anti-mouse IgG, fluorescein isothiocyanate (FITC)-labeled goat anti-rabbit IgG secondary antibodies, and mouse control IgG were purchased from Beyotime. Goat anti-rabbit IgG-horseradish peroxidase (HRP), goat anti-mouse IgG-HRP, and antiubiquitin (anti-Ub) were purchased from Abcam and Sigma, respectively. Tubulin, p53, HUWE1, and TRAF6 antibodies were prepared in our lab.

### Immunofluorescence detection.

Subcellular localization of HUWE1, TRAF6, and p53 in mud crabs was detected via confocal microscopy assay. Mud crab hemocytes were seeded onto a confocal dish and fixed with paraformaldehyde (4%) for 15 min. Then, the hemocytes were permeabilized using saponin (Beyotime, China) for 10 min. After being washed with PBS, the hemocytes were blocked with QuickBlock Western (Beyotime, China) for 30 min at room temperature. Subsequently, the cells were incubated with anti-HUWE1, anti-TRAF6, and anti-p53 antibodies at 4°C overnight, which was followed by washing with PBS and incubation with the Cy3-labeled goat anti-mouse IgG or FITC-labeled goat anti-rabbit secondary antibodies for 1 h at room temperature. Finally, DAPI (4′,6-diamidino-2-phenylindole) was used to stain for the cell nucleus for 5 min at 4°C, and images were obtained with a confocal microscope (Zeiss, Germany).

### Apoptosis measurement by flow cytometric and caspase 3 activity analysis.

An FITC-annexin V apoptosis detection kit (FITC annexin V apoptosis detection kit I; BD Pharmingen, USA) was used to detect the apoptotic rate of hemocytes following the manufacturer’s instructions. Briefly, the collected hemocytes were resuspended in binding buffer and stained with FITC-conjugated annexin V and propidium iodide (PI). Finally, the cells were analyzed by flow cytometry (BD Biosciences, USA). In addition, the apoptotic activity of hemocytes was also evaluated by detecting the caspase 3 activity with a caspase 3 activity assay kit (Beyotime, China). Cells were collected and washed with PBS at 600 × *g* at 4°C for 5 min after being resuspended with lysis buffer for 15 min, and the supernatant was obtained by centrifugation at 16,000 × *g*, followed by incubation with 40 μL of detection buffer and 10 μL of Ac-DEVD-PNA (2 mM) at 37°C for 120 min, and the cells were finally monitored at 405 nm in a microplate reader.

### ROS measurement.

The ROS levels in mud crabs were detected by a reactive oxygen species (ROS) assay kit (Beyotime, China) according to the manufacturer’s instructions. Briefly, mud crab hemocytes were harvested and resuspended in ice-cold acid citrate dextrose (ACD) anticoagulant buffer (1.32% sodium citrate, 0.48% citric acid, and 1.47% glucose) supplemented with 0.1% DCFH-DA (2,7-dichlorodihydrofluorescein diacetate) at 37°C for 20 min. Then, the cells were washed three times with ACD anticoagulant buffer. Finally, a Flex Station II microplate reader (Molecular Devices, USA) was used to examine the ROS levels at excitation/emission (Ex/Em) of 488/525 nm, pictures were captured by fluorescence microscopy (Carl Zeiss, German), and the cell numbers of each sample were counted with an optical microscope and hemacytometer.

### Detection of ubiquitinated protein.

The ubiquitination level of protein was measured by a UbiQapture-Q kit (Enzo, Switzerland) according to the manufacturer’s instructions. After injection with 100 μL of MG132 and PR-619 or equivalent DMSO, mud crab hemocytes were collected and homogenized with cell lysis buffer for Western blotting and IP (Beyotime, China). Then, 40 μL of UbiQapture-Q matrix was added to the cell lysate. Subsequently, the mixture was resuspended gently with the affinity matrix at 4°C overnight. After centrifugation at 5,000 × *g* for 15 s, the matrix was washed with PBS and subjected to Western blotting. Similarly, S2 cells transfected with the indicated plasmids were lysed and used for ubiquitination assay.

### Statistical analyses.

Data are represented as the mean ± standard error of the mean (SEM) from triplicate samples. Statistical analysis was performed by two-way analysis of variance (ANOVA) or Student's *t* test using GraphPad Prism 5.0 (GraphPad Software). *P* values of <0.05 were considered statistically significant.

### Data availability.

RNA-seq data have been uploaded to the NCBI BioProject database under accession no. PRJNA715091.
